# Laboratory markers of disease severity in *Plasmodium knowlesi* infection: a case control study

**DOI:** 10.1186/1475-2875-11-363

**Published:** 2012-10-30

**Authors:** Matthias Willmann, Atique Ahmed, Angela Siner, Ing Tien Wong, Lu Chan Woon, Balbir Singh, Sanjeev Krishna, Janet Cox-Singh

**Affiliations:** 1 Institute of Medical Microbiology and Hygiene, University of Tübingen, Tübingen, Germany; 2 Malaria Research Centre, University Malaysia Sarawak, Kuching, Sarawak, Malaysia; 3 Hospital Sibu, Sibu, Sarawak, 96000, Malaysia; 4 Pathology Laboratory, Hospital Sarikei, Sarikei, Sarawak, 96100, Malaysia; 5 Division of Clinical Sciences, St. George’s, University of London, London, UK; 6 School of Medicine, University of St Andrews, Medical and Biological Sciences Building, North Haugh, St Andrews, KY16 9TF, Fife, UK

**Keywords:** Plasmodium knowlesi, Severity markers, Malaria pigment, Parasitaemia, Platelet count

## Abstract

**Background:**

*Plasmodium knowlesi* malaria causes severe disease in up to 10% of cases in Malaysian Borneo and has a mortality rate of 1 - 2%. However, laboratory markers with the ability to identify patients at risk of developing complications have not yet been assessed as they have for other species of Plasmodium.

**Methods:**

A case control study was undertaken in two hospitals in Sarikei and Sibu, Malaysian Borneo. One hundred and ten patients with uncomplicated (n = 93) and severe (n = 17) *P. knowlesi* malaria were studied. Standardized pigment-containing neutrophil (PCN) count, parasite density and platelet counts were determined and analysed by logistic regression and receiver operating characteristic (ROC) analysis.

**Results:**

The PCN count was strongly associated with risk of disease severity. Patients with high parasite density (≥ 35,000/μl) or with thrombocytopaenia (≤ 45,000/μl) were also more likely to develop complications (odds ratio (OR) = 9.93 and OR = 5.27, respectively). The PCN count yielded the highest area under the ROC curve (AUC) estimate among all markers of severity (AUC = 0.8561, 95% confidence interval: 0.7328, 0.9794). However, the difference between all parameter AUC estimates was not statistically significant (Wald test, p = 0.73).

**Conclusion:**

Counting PCN is labour-intensive and not superior in predicting severity over parasitaemia and platelet counts. Parasite and platelet counts are simpler tests with an acceptable degree of precision. Any adult patient diagnosed with *P. knowlesi* malaria and having a parasite count ≥35,000/μl or ≥1% or a platelet count ≤45,000/μl can be regarded at risk of developing complications and should be managed according to current WHO guidelines for the treatment of severe malaria.

## Background

Human infection with *Plasmodium knowlesi* was thought to be a rare event until an unexpected high incidence of cases was revealed in the Kapit division of Sarawak, Malaysian Borneo in 2004 [1]. Subsequent reports of mixed and mono-infections with *P. knowlesi* from other locations in Sarawak and Sabah, Malaysian Borneo [2], and also from Vietnam, Myanmar, Thailand and the Philippines show a much wider distribution than initially presumed [3-7]. A clinical cohort study on adult patients demonstrated a severity rate of up to 10% and a case fatality rate about 2% [8]. Typical complications include respiratory distress, jaundice and acute renal failure which are features of multiple organ failure also seen in adult *Plasmodium falciparum* infections [9]. In contrast, coma does not appear to be a presenting feature of *P. knowlesi* malaria.

Despite a relatively high rate of complications, markers for identifying *P. knowlesi* malaria patients at risk of severe disease have not been properly assessed. However, higher peripheral blood parasitemia and lower platelet counts are proposed as markers of severity but precise cut-offs are not available [8]. The malaria pigment haemozoin is formed from haem, a toxic byproduct of haemoglobin metabolism by the parasite [10]. As the intra-erythrocytic life-cycle progresses, haemozoin accumulates and becomes visible in late-stage trophozoites and schizonts. Haemozoin is released into the peripheral blood during schizont rupture and is subsequently removed by monocytes and neutrophils [11]. The number of pigment-containing white blood cells (WBC) is a marker of disease severity in *P. falciparum* malaria [11,12]. In severe *P. falciparum* malaria circulating pigment-containing WBCs are significantly higher than in uncomplicated cases or healthy controls and are thought to give a measure of the extent and duration of infection [13-15] but are not a useful predictor of a fatal outcome [16].

The association between parasitaemia, platelets and pigment-containing neutrophils (PCN) and risk of severe disease in *P. knowlesi* infections was investigated here. Cut-offs predictive of the risk of developing severe disease were computed and the relative merit of each as diagnostic tool discussed.

## Methods

### Study site and design

This case–control study was conducted in two hospitals in Sarikei and Sibu, Malaysian Borneo, which have a population of mostly Iban and Chinese ethnicity. Adult patients (≥15 years) admitted between July 2007 and January 2010 were included if they had single-species *P. knowlesi* infection, did not suffer from any significant comorbidity, were not pregnant and had not taken antimalarial treatment within the previous 28 days. At admission, routine haematological testing was performed by using fully automatic Nihon Kohden analyzer (Model 8222, Japan) in Sibu hospital and a Sysmex KX-21 (Japan) or Nihon Kohden analyzer (Model-MEK 6410K, Japan) in Sarikei hospital. Patients with uncomplicated *P. knowlesi* malaria were treated promptly as described elsewhere [8,17]. Patients presenting with or developing features of severe malaria were treated in compliance with WHO guidelines for severe malaria [18]. *Plasmodium* species was confirmed by nested polymerase chain reaction (PCR) assays at the Malaria Research Centre, Universiti Malaysia Sarawak, Kuching, as described previously [1]. Only patients with PCR-confirmed single-species infection with *P. knowlesi* were included in the study. Blood samples, blood films and clinical data were collected following written informed consent. The consent forms and the study protocol have been approved by the Malaysian Ministry of Health’s Medical Research and Ethics Committee. All cases with severe disease were retrospectively identified and included (n = 17). Controls were randomly chosen from the pool of patients with uncomplicated malaria (n = 93).

Classification of severe malaria cases was based on criteria of the World Health Organization (WHO) [18,19]. Hyperparasitaemia at any level was not used as a criterion for severity in this study since an appropriate cut-off for hyperparasitaemia has not yet been assessed for *P. knowlesi* malaria. Moreover, defining a specific cut-off for hyperparasitaemia in *P. knowlesi* malaria was an objective of this study. Uncomplicated cases were defined on the basis of symptoms leading to treatment-seeking behavior in the absence of criteria for severity based on organ involvement. Classification was based on clinical and laboratory parameters gained at hospital admission.

### Malaria pigment assessment

Thin blood films were stained with 10% Giemsa Gurr`s improved R66 (BDH Chemicals LTD, Poole, UK) for 25 minutes after fixation with methanol (Methanol, Merck, Darmstadt, Germany) for 1 min. Peripheral parasite density was determined from thin blood films by counting the number of asexual forms in eight consecutive microscopic fields, assuming 250 red blood cells per field in an area with regular monolayer and 1,000-fold magnification. Results are given in percent infected red blood cells (IRBC). Standard parasite counts of thick blood films were quantified as follows: number of asexual forms per 500 WBCs x actual WBCs/μl/500. If parasitaemia was high, asexual forms per 200 WBCs were counted. Differential counts were conducted manually on thin blood films for each patient.

**Figure 1:**
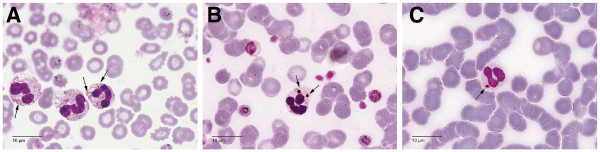
**Giemsa-stained thin blood films.** Amber intracellular haemozoin (arrows) within neutrophils from three different patients with *Plasmodium knowlesi *malaria (**A**-**C**).

For a reliable identification of cell morphology, PCN were detected exclusively on thin blood films by counting 100 neutrophils using an Olympus BX53 microscope with 1000-fold magnification (Figure 1). Subsequently, the percentage of PCN was determined. Two patients were excluded from the study when WBC on the blood films were not identifiable because of poor slide preparation or poor staining. The microscopist was blinded to selection criteria, clinical data and outcome. To investigate intra-observer reliability with regards to the PCN count, 10% of the samples were re-examined by the same microscopist, using another code on the slides. Due to variable WBC counts among the study group, it was necessary to standardize total pigment burden of neutrophils as described previously [13]. Briefly, PCN/μl was calculated as follows: PCN/μl = (number of PCN/100 neutrophils) × (absolute WBC/μl × percent of neutrophils).

### Statistical methods

Baseline characteristics and laboratory findings with numerical outcomes were described on the basis of means and 95% confidence intervals (CI) when data were normally distributed. Hypothesis testing was performed with an unpaired t-test. For skewed data, medians and interquartile ranges were presented. When transformation could achieve normal distribution of values a t-test was subsequently used. Otherwise the Wilcoxon rank sum test was employed. D'Agostino's K-squared test was used to check for normality.

Linear regression analysis was performed on numerical outcomes. Heteroskedasticity was tested by using the Breusch-Pagan and Cook-Weisberg tests and when positive robust standard errors were calculated. Odds ratios (OR) were calculated for categorical variables by using logistic regression. For testing the overall association of categorical variables and risk of severity, a likelihood ratio test was performed. Possible interaction between variables within multivariate logistic regression models was also checked by the use of the likelihood ratio test. Variables were only included into multivariate logistic regression when they were unlikely to lie on the same causal pathway towards the outcome of interest. A possible application of severity markers as diagnostic tools was determined by receiver-operating characteristic (ROC) analyses. For every possible predictor, the area under the ROC curve (AUC) was computed and compared to other predictors by using the Wald test and following an algorithm suggested by DeLong, DeLong and Clarke-Pearson [20]. Since it is necessary in ROC analysis to use a diagnostic rating with increasing values indicating a higher risk, the platelet count could not be directly applied and the inverse function was used. AUC ranges from 0.5 (diagnosis is based simply on chance) to 1 (highest precision). Cut-offs were obtained by examining any value of the ROC curve with regard to its sensitivity and specificity and subsequently predictive values were calculated.

Interclass correlation coefficient was used to assess intra-observer reliability in reading 10% of the thin films by the same microscopist for a second time. A *P* < 0.05 (two-sided) was regarded as statistically significant. All analyses were made by using Stata version 11.0 (Stat Corp., College Station, TX, USA).

## Results

### Demographic characteristics and baseline laboratory findings

A total number of 110 patients were included in the study. According to the WHO criteria for severe falciparum malaria [18,19], 17 patients were classified as severe cases with four fatalities. The most common complication was renal failure (94.1%, Table 1). Demographic features and laboratory findings are listed in Table 2. Both patient groups tend to have a normal WBC count with a slightly higher leukocyte number in patients with severe malaria (p = 0.009). Differences amongst both study groups were noted for potential laboratory markers of severity. The median PCN count was higher in patients with severe malaria (563 *vs* 36 PCN/μl, p <0.0001). Ten per cent of the films were re-examined and an interclass correlation coefficient of 0.7883 for the PCN count was determined. Thick and thin film parasite counts were also significantly different (94256 *vs* 3843.5 parasites/μl, p <0.0001, and 2.5% *vs* 0.3%, p <0.0001). One hundred and seven patients (97.3%) had thrombocytopaenia (<150,000 platelets/μl). However, the maximum platelet count in the study cohort was 167,000 platelets/μl. That indicates a generally low platelet count in all of the patients. Platelet counts were lower in severe *P. knowlesi* malaria compared with uncomplicated disease (38,000 *vs* 69,000 platelets/μl, p = 0.0004).

**Table 1:** Complications of 17 adult patients with severe *Plasmodium knowlesi *malaria

**Complication**	**No. (%)**
Impaired consciousness or unrousable coma	0 (0)
Multiple convulsions – more than two episodes in 24 h	0 (0)
Clinical jaundice plus kidney injury (serum creatinine > 265 μmol/l)	6 (35.3)
Deep breathing, respiratory distress (> 30 breath/min)	3 (17.6)
Circulatory collapse or shock, systolic blood pressure < 70 mm Hg in adults	1 (5.9)
Abnormal spontaneous bleeding	0 (0)
Kidney injury (serum creatinine > 265 μmol/l)	16 (94.1)
Severe normocytic anaemia (packed cell volume < 15%)	1 (5.9)

**Table 2:** **Demographic characteristics and laboratory findings of adult patients with *Plasmodium knowlesi *malaria**

**Variable**	**Uncomplicated malaria n = 93**	**Severe malaria n = 17**	**p-value**
**Demographic characteristic**			
Age, mean (95% CI), years	43.44 (40.4 – 46.5)	49.59 (43.24 – 55.94)	0.11
Male sex, %	68.8	70.6	NA
Iban ethnicity, %	81.7	88.2	NA
**Laboratory findings at admission**			
White blood cell count, x 10^3^/μl^1^	5.9 (5 – 7.55)	8.7 (6.7 – 12.5)	0.009
Neutrophils, mean (95% CI), %	70.4 (68.8 – 71.9)	75.1 (69.9 – 80.4)	0.025
Monocytes, %^1^	2 (1 – 5)	3 (2 – 4)	0.69
Lymphocytes, mean (95% CI), %	25.2 (23.8 – 26.6)	21.2 (16.4 – 26)	0.034
Parasite count, parasites/μl^1^	3843.5 (714.5 – 13030.5)	94256 (18432 – 285768)	<0.0001
Parasite count, % IRBC^1^	0.3 (0.1- 0.7)	2.5 (0.7 – 11.1)	<0.0001
Platelet count, x 10^3^ platelets/μl^1^	69 (44 – 99)	38 (26 – 48)	0.0004
PCN count, cells/μl^1^	36 (0–128)	563 (158 – 1388)	<0.0001

1 median, interquartile range.

95% CI 95% confidence interval.

% IRBC % infected red blood cells.

PCN pigment-containing neutrophils.

NA not assessed.

### Risk assessment of potential laboratory severity markers

A detailed crude risk assessment of potential laboratory severity markers was performed by using logistic regression on different categorical variables (Table 3). The PCN count was strongly associated with risk of severity (p <0.0001). This is particularly accentuated in the group with the highest PCN count (≥416 PCN/μl, OR = 37.58). For analysis of the parasite counts the WHO cut-offs for severe falciparum malaria in low transmission areas were employed (thick film count >100,000 parasites/μl and thin film count >2% IRBC). Both were strongly associated with risk of severity (OR = 9.44 and OR = 8.79, respectively). Furthermore, patients with severe (≤35,000 platelets/μl) and moderate (36,000 platelets/μl – 59,000 platelets/μl) thrombocytopaenia were more likely to have a severe course of disease (p = 0.001).

**Table 3:** **Risk of severe disease among adult patients with *Plasmodium knowlesi *malaria – univariate modeling**

**Variable**		**Uncomplicated malaria n (%)**	**Severe malaria n (%)**	**Crude odds ratio (95% CI)**	**p-value**^**†**^
PCN count (PCN/μl)	0	41 (44.1)	2 (11.8)	1	<0.0001
	1 – 139	32 (34.4)	1 (5.9)	0.64 (0.06 – 7.38)	
	140 – 415	14 (15.1)	3 (17.6)	4.39 (0.66 – 29.06)	
	≥416	6 (6.4)	11 (54.7)	37.58 (6.64 – 212.67)	
Parasite count (parasites/μl)	<100000	85 (91.4)	9 (52.9)	1	0.0003
	≥100000	8 (8.6)	8 (47.1)	9.44 (2.85 – 31.28)	
Parasite count (% IRBC)	<2	80 (86)	7 (41.2)	1	0.0001
	≥2	13 (14)	10 (58.8)	8.79 (2.84 – 27.21)	
Platelet count (x 10^3^ platelets/μl)	≥60	54 (58.1)	2 (11.8)	1	0.001
	36-59	18 (19.3)	8 (47)	12 (2.33 – 61.78)	
	≤35	21 (22.6)	7 (41.2)	9 (1.72 – 46.87)	

95% CI 95% confidence interval.

PCN pigment-containing neutrophils.

% IRBC % infected red blood cells.

^†^All p-values were obtained from likelihood ratio test.

### Linear regression and multivariate logistic regression of potential severity markers

Linear regression was performed on the potential severity markers to investigate associations between them. The results indicate that peripheral parasitaemia measured on thick and thin blood films accounts for 75% and 65% (R^2^ = 0.75 and 0.65 respectively) of the total variation in circulating pigment-containing neutrophils (Table 4). Since both variables might lie on the same causal pathway towards the outcome, they were not included in the same multivariate logistic regression model. However, the variation of the platelet count cannot be explained for the most part by the changes in peripheral parasitaemia (R^2^ = 0.07 and 0.05 respectively) or amount of circulating pigment-containing neutrophils (R^2^ = 0.04) and is thus unlikely to lie on the same causal pathway towards the outcome. To address these issues, three different multivariate models were performed. The results are shown in Table 5. There is some degree of confounding but the platelet count proves to be an independent predictor of severity when corrected for the effects of peripheral parasitaemia and circulating PCN.

**Table 4:** Linear regression analysis of the predictive markers

**Dependent variable**	**Independent variable**	**Coefficient (95% CI)**	**p value**	**R**^**2**^
PCN count (PCN/μl)	Parasite count (% IRBC)	148.19 (95.1 – 201.27)	<0.0001	0.75
PCN count (PCN/μl)	Parasite count (parasites/μl)	0.005 (0.004 – 0.006)	0.0003	0.65
Platelet count (x 10^3^ platelets/μl)	Parasite count (% IRBC)	−2.24 (−3.79 – -0.7)	0.005	0.07
Platelet count (x 10^3^ platelets/μl)	Parasite count (parasites/μl)	−0.00007 (−0.0001 – -0.00001)	0.002	0.05
Platelet count (x 10^3^ platelets/μl)	PCN count (PCN/μl)	−0.01 (−0.19 – -0.0009)	0.031	0.04

95% CI 95% confidence interval.

PCN pigment-containing neutrophils.

% IRBC % infected red blood cells.

R^2^ square of correlation coefficient.

**Table 5:** **Risk of severe disease among adult patients with*Plasmodium knowlesi *malaria – multivariate modeling**

**Model**	**Variables**		**Adjusted OR (95% CI)**	**p value**^**†**^
*Model 1*	Platelet count	≥60	1	
	(x 10^3^ platelets/μl)	36-59	10.37 (1.79 – 59.98)	0.002
		≤35	12.28 (2.04 – 73.86)	
	Parasite count	<100000	1	0.0006
	(parasites/μl)	≥100000	10.74 (2.63 – 43.94)	
*Model 2*	Platelet count	≥60	1	
	(x 10^3^ platelets/μl)	36-59	8.69 (1.59 – 47.48)	0.018
		≤35	5.59 (0.99 – 31.34)	
	Parasite count	<2	1	0.003
	(% IRBC)	≥2	5.97 (1.79 – 19.88)	
*Model 3*	Platelet count	≥60	1	
	(x 10^3^ platelets/μl)	36-59	4.75 (0.74 – 30.36)	0.04
		≤35	9.29 (1.37 – 62.86)	
	PCN count	0	1	
	(PCN/μl)	1 – 139	0.54 (0.04 – 6.63)	<0.0001
		140 – 415	4.8 (0.66 – 34.83)	
		≥416	30.25 (4.74 – 193.22)	

95% CI 95% confidence interval.

PCN pigment-containing neutrophils.

% IRBC % infected red blood cells.

^†^All p-values were obtained from likelihood ratio test.

### Diagnostic precision of severity markers

**Figure 2:**
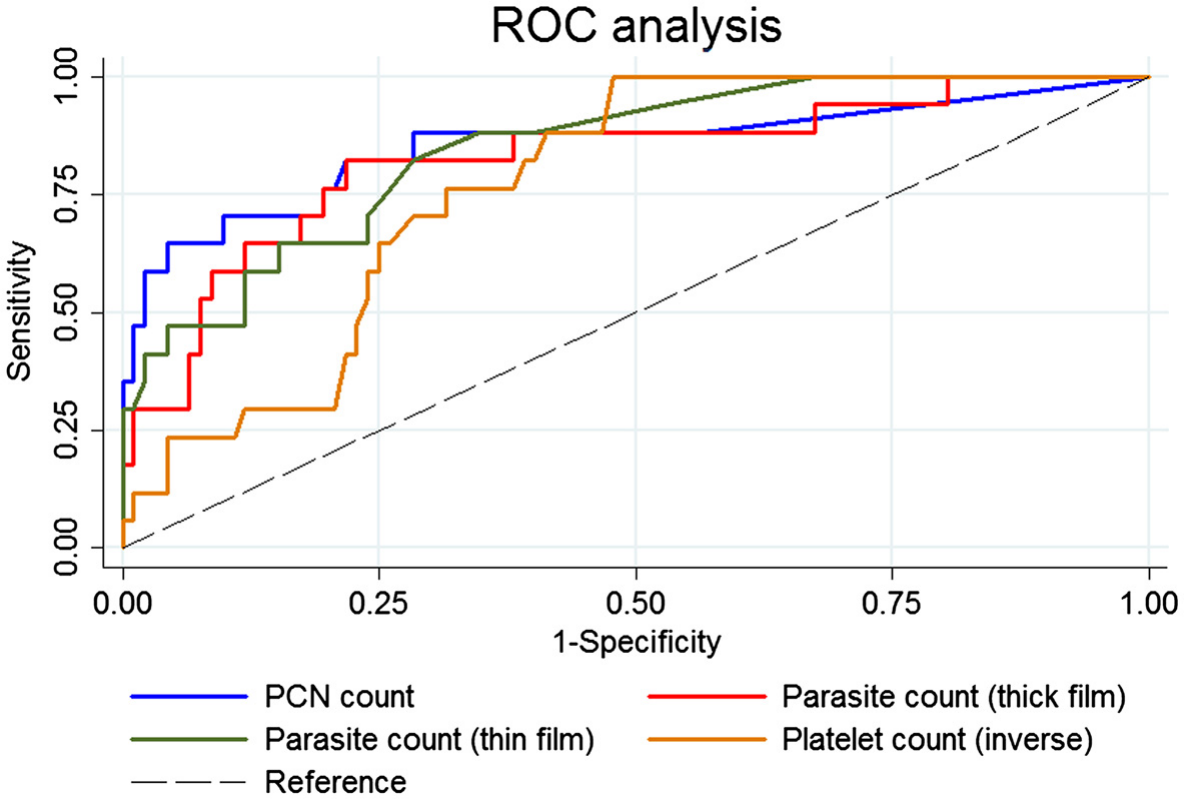
**Area under the ROC curve (AUC) estimates of severity markers.** Receiver-operating characteristic (ROC) analysis revealed no difference in diagnostic potential between all parameters.

A possible diagnostic employment of any marker associated with risk of severity depends fundamentally on its precision to diagnose the outcome of interest. Therefore, ROC analyses were performed on PCN, percent parasitaemia (thin blood film), parasite/μl (thick blood film) and platelet counts as potential laboratory markers of severity as identified during risk assessment (Figure 2, Tables 3 and 5). The PCN count yielded the highest estimate of precision (AUC = 0.8561) whereas the platelet count showed to be the least precise in diagnosing severity (AUC = 0.7723). These results are shown in Table 6. However, despite these distinct estimates there is no evidence that precision differs between PCN, thin and thick film parasite count and platelet count (Wald test, p = 0.73).

**Table 6:** **Receiver operating characteristic (ROC) analysis of severity markers in patients with *Plasmodium knowlesi *malaria**

**Variable**	**AUC (95% CI)**
Pigment-containing neutrophil count in cells/μl	0.8561 (0.7328 – 0.9794)
Parasite count in parasites/μl (thick film)	0.8261 (0.7075 – 0.9447)
Parasite count in % IRBC (thin film)	0.8476 (0.7536 – 0.9416)
Platelet count in x 10^3^ platelets/μl†	0.7723 (0.6775 – 0.8671)

AUC area under ROC curve.

95% CI 95% confidence interval.

% IRBC % infected red blood cells.

NA not assessed.

^†^This analysis was made on the inverse function of the platelet count.

Suitable cut-offs for practical use were evaluated for thick and thin film parasite counts and for platelet counts (Table 7). Candidates for cut-offs were chosen at points of substantial changes in sensitivity and specificity during ROC analysis. Positive and negative predictive values were calculated by assuming a prevalence of 10% severe cases among hospitalized patients with *P. knowlesi* malaria. Such prevalence was reported from a prospective survey in Sarawak, Malaysia [8]. A minimum negative predictive value of 95% was required to be recommended as suitable cut-off. Cut-offs can be used to categorize patients into groups of higher and lower risk of severe *P. knowlesi* malaria. For example, any patient having a parasite count ≥35,000/μl or ≥1% parasitaemia can be regarded to be at high risk of severe disease. Multivariate risk assessment of severity markers when using the recommended cut-offs is shown in Table 8. Both parasite counts are still strongly associated with risk of severity when using the suggested cut-offs instead of the WHO cut-offs of >100,000 parasites/μl or >2% IRBC for *P. falciparum* malaria. Having a platelet count ≤45,000/μl is independently associated with a higher risk of severity (OR = 5.27; p = 0.004).

**Table 7:** Cut-off evaluation of predictive markers

**Variable**	**Cut-off**	**Sensitivity (%)**	**Specificity (%)**	**PPV (%)**	**NPV (%)**	**Odds of severity**
Parasite count (parasites/μl)	≥20000	70.59	79.57	27.74	96.05	0.63
**Parasite count (parasites/μl)**	**≥35000**	**64.71**	**84.95**	**32.33**	**95.59**	**0.79**
Parasite count (parasites/μl)	≥50000	58.82	87.1	33.6	95	0.83
Parasite count (parasites/μl)	≥100000	47.06	91.4	37.81	93.95	1
Parasite count (% IRBC)	≥0.5	88.24	65.59	22.17	98.05	0.47
Parasite count (% IRBC)	≥0.8	70.59	76.34	24.89	95.89	0.54
**Parasite count (% IRBC)**	≥**1**	**64.71**	**78.49**	**25.05**	**95.24**	**0.55**
Parasite count (% IRBC)	≥2	58.82	86.02	31.86	94.95	0.77
Parasite count (% IRBC)	≥3	47.06	89.25	32.72	93.82	0.8
Platelet count (x 10^3^ platelets/μl)	≤60	88.24	55.91	18.19	97.72	0.37
Platelet count (x 10^3^ platelets/μl)	≤50	76.47	68.82	21.41	96.34	0.45
**Platelet count (x 10**^**3**^**platelets/μl)**	≤**45**	**70.59**	**72.04**	**21.91**	**95.66**	**0.46**
Platelet count (x 10^3^ platelets/μl)	≤40	58.82	76.34	21.64	94.35	0.46

% IRBC % infected red blood cells.

PPV positive predictive value.

NPV negative predictive value.

Bolded rows indicate the recommended cut-offs.

**Table 8:** **Risk of severe disease in patients with *Plasmodium knowlesi *malaria using the recommended cut-offs – multivariate modeling**

**Variable**		**Uncomplicated malaria n (%)**	**Severe malaria n (%)**	**Adjusted odds ratio (95c% CI)**	**p-value**^**†**^
Parasite count (parasites/μl)	<35000	79 (71.8)	6 (35.3)	1	0.0001
	≥35000	14 (28.2)	11 (64.7)	9.93 (2.91 – 33.87)	
Parasite count (% IRBC)	<1	73 (78.5)	6 (35.3)	1	0.003
	≥1	20 (21.5)	11 (64.7)	5.72 (1.78 – 18.37)	
Platelet count (x 10^3^ platelets/μl)	>45	67 (72)	5 (29.4)	1	0.004
	≤45	26 (28)	12 (70.6)	5.27 (1.59 – 17.41)*	

95% CI 95% confidence interval.

% IRBC % infected red blood cells.

^†^All p-values were obtained from likelihood ratio test.

*The odds ratio was obtained by including the platelet count into one model with parasite count (% IRBC) measured on thin blood films.

## Discussion

Since *P. knowlesi* malaria causes severe disease in approximately one of 10 patients and can be potentially fatal [8], the search for reliable and unsophisticated markers of disease severity that complement the diagnosis of malaria needs to be intensified. The objectives of the study presented here were to test for an association between pigment-containing neutrophil counts and risk of disease severity and to compare with parasitaemia and platelet counts as predictors of severe *P. knowlesi* malaria. Pigment-containing monocytes are also a marker of severe *P. falciparum* malaria [12]. However, due to the low number of monocytes found in thin blood films, a count of 100 monocytes is highly time consuming and was considered as an impractical procedure for routine use and was therefore not performed here.

A strong association between PCN count and risk of disease severity was observed. The method involved counting 100 neutrophils, took 15–20 minutes and requires skilled personal to identify the correct cell morphology. It is both labour-intensive and time consuming in the routine malaria diagnostic setting. Therefore, the PCN count should have a superior prognostic ability over parasitaemia and platelet counts if it is to be recommended for routine use. Even though the AUC estimate for PCN was the highest among all parameter estimates, PCN counts did not have a higher diagnostic precision when compared with parasite and platelet counts. The lack of statistical significance might be explained by the low sample size. On the other hand, linear regression analysis revealed that the total variation in the amount of pigment-containing neutrophils can be explained by the changes in peripheral parasitaemia to an extent of up to 75%, leaving only a minor proportion in the total variation that might represent sequestration and would give additional prognostic information in *P. knowlesi* malaria. In any case, the observed differences in the AUC estimates are rather small and cannot justify a recommendation of the PCN counts as a routine test at the moment. Flow cytometry can achieve a fast and effective detection of pigment-containing WBCs [15,21] but remains a future approach in most settings.

In contrast, the parasite count on thick and thin blood films is a simpler method and is usually available in resource-poor settings. The peripheral blood parasitaemia on thick blood films has been shown to be a potential marker of severity in *P. knowlesi* malaria [8]. The authors of the same study revealed an inverse correlation between parasitaemia and platelet count and suggested that the latter could also be a predictive factor. The number of severe cases in that study was small and the findings were confirmed here, even if the platelet count seems to have a lower accuracy in predicting severe disease when compared with PCN counts and parasitaemia.

Assessing cut-offs with a more predictive than reflective capability was based on an appropriate relationship between positive and negative predictive values. Recommendation of a specific cut-off required a negative predictive value of at least 95% in the study. This would mean that one in 20 patients with a test result indicating non-severe disease or a low risk of developing complications would have severe disease at the time of measurement. None of the severity markers could reach high positive predictive values in the study, assuming a prevalence of 10% severe cases among hospitalized patients with *P. knowlesi* malaria. A positive predictive value of about 33% as calculated for the recommended cut-off of 35,000 parasites/μl means that two of three patients with a positive test indicating a severe disease or a high risk of developing complications would not suffer from severe malaria at the time of measurement. Managing them according to the WHO guidelines for severe malaria might appear as an overtreatment and potential waste of resources in an often resource-limited setting. However, when using the recommended cut-offs in multivariate logistic regression analysis any tested severity marker is independently associated with high risk of severity (OR 5.27 – 9.93). Therefore, having a positive test result in accord with the recommended cut-offs categorizes a patient to be at high risk of severity regardless of the actual clinical status. Management for severe malaria should consecutively be accomplished to prevent a potential development of severe disease in patients within this high-risk group even if they do not suffer from complications at the time of measurement. Consequently, the results of the study presented here indicate that any adult patient diagnosed with *P. knowlesi* malaria and a parasite count ≥35,000/μl or ≥1% or a platelet count ≤45,000/μl should be regarded at risk and should subsequently be treated as for severe malaria according to current WHO guidelines.

Using these cut-offs in areas with prevalence of both *P. knowlesi* and *Plasmodium malariae* may be an issue. *Plasmodium knowlesi* is often confused with *P. malariae* when making the diagnosis by light microscopy [22]. *Plasmodium malariae* infection causes a median parasitaemia of 8,875 parasites/μl with maximum of about 50,000 parasites/μl [23], but rarely causes severe disease. *P. knowlesi* may be wrongly diagnosed in the presence of a *P. malariae* infection. In such cases, a parasite count ≥35,000/μl alone may lead to incorrect risk estimation although coupled with low platelet counts may be more specific. In Sarawak and Sabah, *P. malariae* infection is rare [2,24] and the benefit of properly diagnosing *P. knowlesi* would override misdiagnosing *P. malariae* in a small number of cases.

One major limitation of this study is the assumption that severe cases of *P. knowlesi* malaria occur with a prevalence of 10% among hospitalized patients. The prevalence and characteristics of *P. knowlesi* malaria should be measured within the framework of prospective studies in all locations in Southeast Asia where *P. knowlesi* malaria occurs [3-6] and where validation of the findings presented here would be informative. Cut-offs might be adjusted according to the results gained in such studies. The analyses conducted here identify parasitaemia and platelet counts as measures of risk assessment for the development of severe disease in *P. knowlesi* infections. However, additional simple diagnostic tools to distinguish *P. knowlesi* from *P. malariae* infections in settings with limited resources are urgently needed to identify patients at risk of severe *P. knowlesi* malaria.

## Conclusion

Based on the results obtained, parasite and platelet counts are precise tests for indentifying patients at risk of severe *P. knowlesi* malaria. Counting PCN is not superior in predicting severity but labour-intensive and can therefore not be recommended for routine diagnosis of *P. knowlesi* infections.

## Competing interests

The authors declare that they have no competing interests.

## Authors’ contribution

MW, AA, AS, ITW and LCW collected the blood samples and patients’ data. MW, AA and AS carried out the laboratory work. MW participated in the design of the study and analysed the data. JCS, BS and SK conceived the study and participated in its coordination. MW and JCS wrote the manuscript. All authors read, commented on and approved the final manuscript.
